# Facial features and unethical behavior – Doped athletes show higher facial width-to-height ratios than non-doping sanctioned athletes

**DOI:** 10.1371/journal.pone.0224472

**Published:** 2019-10-30

**Authors:** Bjoern Krenn, Callum Buehler

**Affiliations:** Department of Sports Sciences, University of Vienna, Vienna, Austria; University of Jyväskylä, FINLAND

## Abstract

Past research has emphasized the role of facial structures in predicting social behavior. In particular the facial width-to-height ratio (fWHR) was found to be a reliable predictor for antisocial and unethical behavior. The current study was aimed at examining this association in the field of sports: FWHRs of 146 doping sanctioned athletes in athletics (37 male/38 female) and weightlifting (44 male/27 female) were compared to the fWHRs of randomly chosen non-doping sanctioned athletes of the Top Ten at the World Championship 2017 and Olympic Games 2016 in both sports (146 athletes). The results showed that doping sanctioned athletes due to the use of anabolic steroids had larger fWHRs than non-doping sanctioned athletes. However, doping sanctioned athletes due to other doping rule violations than the use of anabolic steroids, did not show this effect. The study provides empirical evidence for the relation between fWHR and unethical behavior in a real-world setting and contributes to the discussion about fWHR’s biological origin, emphasizing the role of anabolic steroids. A mutual interaction between fWHR and doping behavior is discussed, at which a larger fWHR might signify a higher tendency to behave unethically, whereas the consequential intake of anabolic steroids might also shape individuals’ faces.

## Introduction

Past research revealed that human faces serve as a crucial source of information for others, affecting social interactions [1, 2]. Variable as well as static facial features were reported to be meaningful and predictive for human behavior [3, 4]. Recently, a growing body of research has emphasized the role of the facial width-to-height ratio (fWHR). The fWHR is calculated by dividing the distance between the left and right zygion by the distance between the nasion and prosthion [5]. Past research documented positive correlations between individual’s fWHR and perception of aggression, dominance and threat by others [6–9]. Moreover, research found empirical evidence that fWHR also predicts behavioral aspects: Individuals with a higher fWHR were found to show a higher tendency of acting aggressively, threatening and dominant in a broad range of different situations and societal contexts [10–19].

However, although meta-analyses found a small but robust link between fWHR and aggressive behavior as well as dominance [10, 16], several studies were unable to comprehensively corroborate previously reported findings of fWHR [20–23]. As a consequence, concerns were raised about the replicability of published fWHR findings [20, 21]. This tendency was further increased by the fact that the mechanism determining an association between fWHR and behavioral facets is still not well understood: It was hypothesized that testosterone is the mutual origin affecting both fWHR and behavioral aspects (e.g. aggressive behavior) [5, 15]. However, this hypothesis turned out to be contentious, and clear empirical evidence is still missing [24, 25]. Neither the circulating testosterone level, nor the prenatal testosterone exposure stably and reliably predicted an individual’s fWHR [25–28]. In addition, results analyzing the role of pubertal testosterone in determining individual’s fWHR differed between the studies [24, 26, 28, 29]. Thus, research into the underlying mechanism forming the association between fWHR and its behavioral correlates has remained fragmented.

Following the link between an individual’s fWHR and aggressive and dominant behavior, research also revealed fWHR to be associated with unethical and antisocial behavior. Empirical evidence was found that persons with a higher fWHR more readily use explicit deception, cheat and exploit the trust of others for their personal gain than those with a smaller fWHR [11, 12, 30]. The current study tied in with this research and questioned a link between fWHR and unethical behavior in a real world setting: We investigated fWHR’s role in doping behavior in the field of high performance sports. Taking the code of ethics and the main principle of fairness in sports into consideration, doping represents a clear example of unethical and antisocial behavior [31, 32]: The decision to use performance-enhancing drugs signifies an illegal act of the moral intent to take an unfair advantage over an opponent [31– 34]: Thus, we compared fWHR between doping-sanctioned and non-doping sanctioned athletes in weightlifting and athletics. Both sports represent disciplines among the highest frequencies of detected doping violations [31]. We predicted that doping sanctioned athletes would have higher fWHRs than non-doping sanctioned athletes in both sports. Taking the assumed link between fWHR and testosterone into consideration, we additionally differentiated between doping sanctioned athletes due to the use of testosterone and anabolic steroids and those sanctioned due to the other doping rule violations. Potential differences between these groups might enable deeper insights into the origin of varying fWHRs and its correlates.

The literature suggests that the correlation between higher fWHR and unethical and antisocial behavior only occurs in men, but not in women [11, 12, 30]. As the potential reason for the sex differences, the underlying differential exposure to testosterone and anabolic steroids through certain phases of individuals’ development was argued [5, 6, 15]. However, research failed to detect a solid empirical base in line with this assumption. Even when several studies argued fWHR to be sexually dimorphic [5, 15, 16], a range of studies lacked in empirical evidence of significant differences between men and women [30, 35–36]. Taking the assumed link of testosterone and fWHR into consideration, female dopers might bring additional insights into the underlying mechanism of varying fWHR, as testosterone and anabolic steroids are the most common doping substances. Previous research stated that in male delayed puberty adolescents, exogenous testosterone administration significantly enhanced craniofacial growth [37]. Also, in female-to-male transsexual persons, the administration of testosterone was accompanied by qualitative and quantitative facial changes: Most notably, the total face was widened [38]. It can be assumed that the effects of exogenous testosterone on facial changes would be intensified in females, taking the 20-30-fold lower concentrations of endogenous testosterone in women than men into consideration [27, 39–42]. This has already been documented in animal studies [43–45]. As a consequence, we particularly expected doping sanctioned women due to the use of testosterone and anabolic steroids to show larger fWHR than doping-sanctioned women due to other doping rule violations as well as non-doping sanctioned female athletes.

## Material and methods

Lists of doping sanctioned athletes were obtained via the International Weightlifting Federation (www.iwf.net) and the Athletes Integrity Unit (authority for the management of integrity programs of the International Association of Athletics Federation; www.athletesintegrity.org). In weightlifting we considered all athletes that were suspended sometime between 2004 and 2017 due to doping violations. In athletics, all currently sanctioned athletes as of the 05/30/2018 were taken into consideration. All detected substances leading to athletes’ doping suspensions were registered. In order to measure fWHR, photographs were obtained via Google’s image search. Only forward-facing images were selected, precluding any noticeable effects of different head tilts and positions [46–48]. Two independent research assistants controlled for neutral facial expressions and visibility of both zygions in order to take more care of the disturbance variables in fWHR measurements due to facial expressions and camera/head positions [47, 49]. A total of 75 athletes in athletics (37 male/38 female) and 71 (44 male/27 female) in weightlifting were found. To generate a control group for the comparison to doping sanctioned athletes, the results of the World Championships in 2017 were analyzed in both sports. In weightlifting, the Top Ten at the Olympic Games in 2016 were also screened for usable data, because the frequencies of doped athletes exceeded the number of the remaining athletes in specific weight classes. For each doping sanctioned athlete, we randomly selected one athlete of the same discipline in athletics, or weight class in weightlifting. Former doping sanctioned but reinstated athletes were not considered in the control groups.

FWHRs were measured using the software ImageJ (NIH open-source software) in accordance with the instructions made by Weston et al. [5] and Carré and McCormick [15]. FWHR’s measurement reliability of two independent measurers was high (.93) and comparable with previous studies [21, 22]. FWHR’s mean of both measurers was calculated and incorporated in the all statistical analyses. In addition, fWHR’s within subject repeatability was calculated for 28 athletes, for which two photographs—following the mentioned selection criteria—were found: Correlation coefficients for both measurers turned out satisfactorily (*r* = .89/.92) and the means revealed stable (*M*_*A*_ = 2.08 ± .17; *M*_*B*_ = 2.09 ± .18) indicating an acceptable repeatability.

To analyze potential differences in the fWHR an univariate ANOVA for all athletes was conducted. At first, sex, sports (weightlifting vs. track & field) and doping behavior were incorporated as predictor variables. To enable us to take a closer look at differing effects of doping substances, we differentiated between athletes sanctioned for anabolic steroid doping (*n* = 112) and those who were sanctioned due to other doping rule violations (*n* = 34; cf. [50]). In our doping sanctioned sample of athletics, 49 athletes (65%) were sanctioned due to the use of anabolic steroids, whereas 26 athletes (35%) were suspended due to violations of other doping rules. In weightlifting the percentage of athletes, who were suspended due to the use of anabolic steroids was relatively high (89%). Only seven male weightlifters and one female weightlifter were sanctioned for violations of differing doping rules.

In the final step, we focused more intensively on the similarities and differences within the diverse athletic disciplines (cf. [22]). Thus, we executed a univariate ANOVA incorporating the sport categories of weightlifting, throwing disciplines (e.g. pole vault), sprint disciplines (e.g. 110 meters hurdles), and endurance disciplines (e.g. marathon) instead of sport type (weightlifting vs. track & field athletes). As a consequence, eight track and field athletes had to be excluded in this analysis due to the small sample size of jumping disciplines (*n* = 6) and heptathlon (*n* = 2). For all analyses the software IBM SPSS Statistics (Version 21.0) was used. Alpha level was set at .05 in all analyses. Post-hoc analyses were conducted via Bonferroni tests. A potential impact of body weight [51] on fWHR was examined in the subsample of weightlifters’ only via their corresponding weight class. In track and field athletes this data was not accessible. Body weight’s impact was revealed as significant in the sample of weightlifters, whereas it did not affect the significance of the subsequent findings. Thus, we did not report these results within the manuscript, but reported them in a supplementary file (S1 File).

## Results

The conducted ANOVA revealed a significant effect of doping (*F*(2, 280) = 7.45, *p* < .001, η^2^ = .05), and sports type (*F*(1, 280) = 9.79, *p* = .01, η^2^ = .03). The impact of sex (*F*(1, 280) = .25, *p* = .62) as well as the interactions of doping and sex (*F*(2, 280) = 2.49, *p* = .09), doping and sports type (*F*(2, 280) = .20, *p* = .82), sex and sports type (*F*(2, 280) = .06, *p* = .80) did not reveal any significance. Bonferroni post hoc tests clarified that doping sanctioned athletes due to the use of anabolic steroids showed significantly larger fWHRs than non-doping sanctioned athletes (*p* < .001). The differences between doping sanctioned athletes due to the use of anabolic steroids and doping sanctioned athletes due to other doping rule violations did not turn out to be significant (*p* = .62), nor did the difference between non-doping sanctioned athletes and doping sanctioned athletes due to other doping rule violations than anabolic steroids (*p* = .57). Concerning the significant impact of sport type, it was revealed that weightlifters showed a larger fWHR than track and field athletes. Table 1 shows the means and standard deviations of each subsamples’ fWHR.

**Table 1:** Facial width-to-height ratio (fWHR) for doping and non-doping sanctioned athletes.

	Doping sanctioned athletes’ fWHR						Non doping sanctioned athletes’ fWHR		
	Anabolic steroids			Other doping rule violations					
	*n*	*M*	*SD*	*n*	*M*	*SD*	*n*	*M*	*SD*
Athletics	49	1.97	.13	26	1.95	.13	75	1.90	.16
Weightlifting	63	2.06	.18	8	2.10	.14	71	1.99	.16
Total	112	2.02	.16	34	1.98	.15	146	1.94	.16

In the second step, a univariate ANOVA was conducted incorporating sport disciplines (weightlifting, throwing, endurance & sprinting) instead of sport types. Again the impact of doping substance (*F*(2, 261) = 4.04, *p* = .01, η^2^ = .03) as well as the impact of sport discipline (*F*(3, 261) = 8.02, *p* < .001, 03B7^2^ = .08) turned out to be significant. Neither the main effect of sex (*F*(1, 261) = .08, *p* = .77), nor the interactional terms of doping and sport discipline (*F*(6, 261) = .61, *p* = .72), doping and sex (*F*(2, 261) = 2.37, *p* = .10) as well as sport discipline and sex (*F*(3, 261) = .26, *p* = .85) revealed statistical significance. Bonferroni post hoc tests again clarified larger fWHRs for doping sanctioned athletes due to the use of anabolic steroids than non-doping sanctioned athletes (*p* < .001). The differences of doping sanctioned athletes due to other doping rule violations to non-doping sanctioned athletes (*p* = .55) but also to doping sanctioned athletes due to the use of anabolic steroids (*p* = .63) did not reveal significance. Concerning sport disciplines athletes of weightlifting (*M* = 2.02 ± .17) and throwing disciplines (*M* = 2.02 ± .13) showed lager fWHRs than athletes of endurance (*M* = 1.90 ± .14; weightlifting: *p* < .001; throwing disciplines: *p* < .001) and sprinting disciplines (*M* = 1.88 ± .13; weightlifting: *p* < .001; throwing disciplines: *p* < .001).

## Discussion

The current study questioned the relationship between fWHR and antisocial and unethical behavior in the real world setting of sports. We compared non-doping sanctioned athletes with doping sanctioned athletes due to the use of anabolic steroids as well as doping sanctioned athletes due to other doping rule violations in athletics and weightlifting. We revealed empirical evidence for a significant relationship between fWHR and unethical behavior: Athletes, who were suspended due to intake of anabolic steroids showed larger fWHRs than non-doping sanctioned athletes. This effect was revealed in both sports and tied in with the findings of experimental studies documenting an association between larger fWHR and the tendency to use explicit deception, to cheat or to exploit the trust of others for personal gain [11, 12, 30].

However, the effect size was low and the difference between doping sanctioned athletes, who violated other doping rules than the use of anabolic steroids and non-doping sanctioned athletes did not reveal significance. Also, sex did not affect the impact of doping substance on athletes’ fWHR. In the current data we did not find any empirical evidence of fWHR to be sexually dimorphic [22, 30, 35–36]. Our findings rather suggest an impact of the intake of anabolic steroids on athletes’ fWHR. Thus, the enlarged fWHR of anabolic steroid dopers would rather be understood as a medium- to long-term consequence of the use of anabolic steroids than as an antecedent of unethical behavior. In this regard, previous research already documented an increased facial growth due to the intake of anabolic steroids [37–45]. This might also explain why we did not detect any significant differences in fWHR between female and male athletes: It was argued that the effects of anabolic steroids on facial changes to be intensified in females, taking the 20-30-fold lower concentrations of endogenous testosterone in women than men into consideration [27, 39–42]. However, we also did not detect any fWHR differences between female and male non-doping sanctioned athletes.

The results also showed fWHR differences between sport disciplines. The findings clarified larger fWHRs for weightlifters and athletes of throwing disciplines (athletics) than for athletes of endurance and sprinting disciplines (athletics). In line with the findings of Kramer [22] it seems that this result might be attributed to body size and body weight differences between sport categories. Weightlifters and athletes of throwing disciplines like discus or vault traditionally, need more body mass to reach maximum performances than their counterparts of sprinting and endurance disciplines, at which enlarged body mass might also present additional weight to carry. In this regard, previous research already showed significant correlations between individual’s weight and fWHR [36, 51].

However, due to the highly selective nature of our sample (elite athletes) the generalizability of our findings to the general population is restricted [22]. Further. it has to be considered, that the control group of non-doping sanctioned athletes was composed of successful athletes (Top Ten at the World Championship/Olympic Games), who did not violate any doping rules at the time of the data collection. Thus, the reported findings are limited due to the fact that our control group may also contain dopers that were undetected during the competition's controls. However, this potential impact should rather lessen the differences between the groups of doping sanctioned and non-doping sanctioned athletes, which might induce an even larger effect when factually non-doped athletes are analyzed. In addition, previous research suggested fWHR to be correlated positively with success in several sports [13, 44], which in turn might emphasize the significance of our finding, considering these highly competitive and successful control groups.

Our research remained limited in not clarifying if fWHR affected doping behavior or whether anabolic steroids affected an individual’s fWHR. Our results suggest a mutual interaction between individual’s fWHR and doping behavior, at which a higher fWHR might signify a higher tendency to behave unethically, whereas the consequential intake of anabolic steroids might also shape individuals’ faces. Future research is challenged to cast more light on the foundation of this interaction. Most notably, taking into consideration that these findings might also indicate an association between fWHR and dominance and/or achievement drive [7–10, 14–16]. Following this idea, an enhanced drive to dominate others and to be successful in competitions might enhance the tendency to accept unfair practices and methods to reach one’s targets. In this regard, a significant interaction between fWHR and unethical behavior might also display a consequence of a direct association between dominance and fWHR [5, 6, 15, 19]. However, it also seems possible that athletes with larger fWHRs do appear more distrustful to others, and–as a consequence–are investigated for doping more often, which should also increase the likelihood of being caught. Further research has to take a closer look at these alternative explanations.

In conclusion, the current study provided evidence for the link between fWHR and unethical behavior by analyzing behavioral data outside of the laboratory: Anabolic steroid-doped athletes showed larger fWHRs than non-doping sanctioned athletes. However, the validity of the reported findings is limited due to small sample sizes, the measurements of fWHR in archival photographs [46, 47] and limited access to potential moderating variables [11, 19, 22, 51]. Future research is required to address these limitations. The results provide additional insights emphasizing the role of anabolic steroids in shaping athletes’ fWHR. A mutual interaction of testosterone and anabolic steroids with unethical and antisocial behavior displays an explanation of our findings.

## Supporting information

S1 File Comparison of the results of the ANOVA and ANCOVA (incorporating weight class as covariate) in the subsample of weightlifters (dependent variable: Athletes’ fWHR). (DOCX)

S2 File Data set including all variables considered in the data analysis. (SAV)
