# Supplementary material for: Facial features and unethical behavior – Doped athletes show higher facial width-to-height ratios than non-doping sanctioned athletes
Source: PLoS One. 2019 Oct 30;14(10):e0224472. doi: 10.1371/journal.pone.0224472 (PMC6821090; doi:10.1371/journal.pone.0224472)
Supplement: S1 File — (DOCX) [file pone.0224472.s001.docx]

**Supplementary file**

To consider the potential impact of athletes’ body weight on fWHR and the reported findings, we conducted an univariate ANCOVA incorporating the weight class of the weightlifters as covariate. The results of the ANOVA and ANCOVA (including athletes’ weight class as covariate) are presented below. The ANCOVA results clarify a significant impact of athletes’ weight class on fWHR. However, the significant impact of doping substance still remained when weight class was incorporated in the model.

Table S1. Results of the ANOVA and ANCOVA (incorporating weight class as covariate) in the subsample of weightlifters for their fWHR.

|  |  | *df* | *F* | *p* | ɳ^2^ | |
| --- | --- | --- | --- | --- | --- | --- |
| ANOVA |  |  |  |  |  | |
|  | Doping | 2;136 | 3.72 | .03 | .05 | |
|  | Sex | 1;136 | .15 | .70 | .00 | |
|  | Doping*Sex | 2;136 | 1.37 | .26 | .02 | |
| ANCOVA |  |  |  |  |  | |
|  | Doping | 2;135 | 4.05 | .02 | .06 | |
|  | Sex | 1;135 | .06 | .80 | .00 | |
|  | Doping*Sex | 2;135 | 1.31 | .27 | .02 | |
|  | Weight class | 1;135 | 9.07 | .01 | .06 |  |
